# Salivary levels of amyloid beta reflect brain amyloid beta burden in cognitively-normal older adults

**DOI:** 10.1016/j.tjpad.2025.100216

**Published:** 2025-06-09

**Authors:** Alison R. Bamford, Jenna N. Adams, Soyun Kim, Lisa M. Taylor, Nandita Tuteja, Liv C. McMillan, Negin Sattari, Ivy Y. Chen, Miranda G. Chappel-Farley, Yuritza Escalante, Alyssa L. Lawrence, Novelle J. Meza, Destiny E. Berisha, Abhishek Dave, Rond Malhas, Mark Mapstone, Bryce A. Mander, Michael A. Yassa, Elizabeth A. Thomas

**Affiliations:** a Department of Neurobiology and Behavior, University of California Irvine, Irvine, CA, USA; b Institute for Interdisciplinary Salivary Bioscience Research, University of California Irvine, Irvine, CA, USA; c Center for the Neurobiology of Learning and Memory, University of California Irvine, Irvine, CA, USA; d Department of Psychiatry and Human Behavior, University of California Irvine, Irvine, CA, USA; e Department of Psychiatry, University of Pittsburgh School of Medicine, Pittsburgh, PA, USA; f Department of Neurology, School of Medicine, University of California Irvine, Irvine, CA, USA; g Department of Cognitive Sciences, University of California Irvine Irvine, CA, USA; h Institute for Memory Impairments and Neurological Disorders, University of California Irvine, Irvine, CA, USA; i Department of Neurosciences, The Scripps Research Institute, La Jolla, CA, USA

**Keywords:** Alzheimer’s disease, amyloid beta, positron emission tomography, neurodegeneration, saliva, biomarker

## Abstract

**Background:**

Amyloid beta (A*β*) plaque burden, as measured by positron emission tomography (PET), is increasingly being used as a biomarker for Alzheimer’s disease (AD) as well as a screening or monitoring tool for clinical trials with amyloid-lowering drugs. However, PET imaging is expensive, invasive and not widely available for all patients, necessitating alternative means to assess brain A*β* accumulation.

**Objectives:**

In this study, we measured levels of A*β*42, A*β*40 and A*β*38 in saliva samples from cognitively unimpaired older adults (n=93; 61.7 % female; mean age = 70.1 ± 6.6 years) using the Mesoscale Discovery platform, carefully considering preanalytical variables, including timing of sample collection, blood contamination and sample concentration. We next determined the relationships between A*β* peptide levels and A*β* plaque burden within the brain, determined using 18F-florbetapir (FBP) PET.

**Results:**

We found that salivary levels of A*β*38 and A*β*42, but not A*β*40 nor the A*β*42/A*β*40, were significantly positively correlated with the global mean FBP standardized uptake value ratio (SUVR), before and after adjusting for age, sex and time of day of saliva sample collection (r=0.523/0.544, p=0.001/0.002 and r=0.316/0.32, p=0.031/0.044, for A*β*38 and A*β*42, respectively). Similar results were observed when A*β* values were analyzed as a ratio to the total protein levels in each sample and when tested in saliva samples that were collected during a restricted morning time window. Using composite regions which represent cortical regions vulnerable to A*β* accumulation in early, intermediate, and late stages of AD, we found that A*β*38 showed the most robust correlation with FBP SUVRs from early-accumulating brain regions (r=0.510; p<0.001). In contrast to the observed effects in saliva, plasma levels of A*β*42 measured from a subset of the participants showed a significant negative correlation to mean FBP SUVR. Using logistic regression analysis to determine whether any salivary A*β* species could predict brain A*β* burden, we found that salivary levels of A*β*38 in combination with age, sex, sample timing and *APOE* genotype could predict A*β*-PET positivity with an area under the curve = 0.950 (95 % confidence interval, 0.876–1.0; p<0.0001).

**Conclusions:**

Our findings suggest that salivary A*β*38 and/or A*β*42 could have relevance as a non-invasive, and more widely applicable biomarker, for utility in clinical studies on AD.

## 1. Introduction

The global burden of people living with dementia and Alzheimer’s disease (AD) has more than doubled in the past two decades, largely due to increased lifespan and population growth [1]. This increase presents a challenge to health-care systems worldwide, necessitating innovative approaches for disease prevention and cost-effective and decentralized diagnostic screening.

Detecting amyloid beta (A*β*) pathology early in the disease process has become increasingly important for the diagnosis and management of AD, as well as for the design of clinical trials for AD. With the development of anti-amyloid agents, such as aducanumab, lecanemab, and donanemab [2–4] as putative disease-modifying treatment options, one of the major challenges is to identify patients with A*β* plaques, especially early in the disease process. Although A*β* positron emission tomography (PET) imaging and cerebrospinal fluid (CSF) biomarkers are known to reflect the presence of A*β* plaques in the brain, these methods are expensive and invasive [5] and not widely accessible for many populations, including low-income individuals or those living in rural communities. The development of blood-based biomarkers that can predict A*β* positivity represents a critical advancement in biomarker discovery; however, blood sampling remains an invasive technique with many drawbacks. Saliva represents an alternative biofluid that has been growing in attention in recent years. One of the main advantages of saliva as a biofluid is that it is easy to collect in a non-invasive way, which can reduce discomfort and anxiety for the individual, especially in elderly populations. Further, compared to blood sampling, whole saliva collection requires no specially trained personnel and does not need to be processed immediately allowing for sample collection in any setting, including the home.

Saliva as a biofluid has been explored for many neurological disorders, including AD [6]. Notably, many past studies have quantified levels of A*β*40 and A*β*42, two of the most widely studied AD biomarkers, in saliva samples. While early studies were not able to detect A*β*42 in saliva samples, due to methodology and saliva collection methods [7,8], many recent studies using sensitive immunoassays and whole saliva collection techniques have reported increased levels of A*β*42 in AD patients [9–15], although one study reported a slight decrease in AD patients compared to normal controls [16]. One drawback of these past studies on saliva is the lack of standardization in saliva collection protocols used and the lack of consideration of potential confounds to saliva biomarker measurements, which include diurnal variation or time of sample collection, saliva sample contamination as well as potential effects of dehydration or concentrated samples. Further, no previous studies have quantified levels of salivary A*β*38 peptide, a widely understudied shorter A*β* species with possibly improved biomarker potential in AD [17,18], nor investigated associations between salivary A*β* peptides and PET-assessed A*β* plaque burden.

To further explore the utility of salivary A*β* peptides as AD biomarkers, in this study, we quantified levels of A*β*42, A*β*40 and A*β*38, in saliva samples from cognitively unimpaired older adults and investigated associations among salivary and plasma A*β* species and A*β*-PET measures. Next, we investigated the effects of preanalytical variables on salivary A*β* measures, in order to help define standardized procedures for the use of saliva as a biofluid for biomarker research. Our findings suggest that salivary A*β*38, and possibly A*β*42, may represent non-invasive biomarkers that can accurately predict A*β* plaque burden.

## 2. Methods

### 2.1. Participants

Participants were recruited from two studies, the Biomarker Exploration in Aging, Cognition, and Neurodegeneration (BEACoN) and an independent sleep study, conducted at the University of California, Irvine. For both studies, inclusion criteria consisted of being 60–85 years old, fluency in spoken English, visual and auditory acuity adequate to complete cognitive assessments, and generally normal cognition, defined by a Clinical Dementia Rating scale of 0 [19]. The sleep study had additional inclusion criteria, including a Mini-Mental State Examination score > 26 and a Functional Assessment Staging Tool (FAST) of Stage 1 or 2 [19]. Participants from both studies were excluded if they had a history of significant co-morbid neurological or psychiatric conditions, major medical conditions that significantly affect cognition, a diagnosis of mild cognitive impairment, dementia or other cognitive impairment, or history of alcohol or substance use disorders within the last two years. Participants were also excluded from the sleep study if they were actively being treated for a sleep disorder, had traveled across >3 hours of time zones within three weeks of the study, had consumed caffeine after 9 AM on the day of the sleep study or were taking non-SSRI antidepressants, neuroleptics, chronic anxiolytics, or sedative hypnotics. All experimental protocols were approved by the Institutional Review Board (IRB) of the University of California, Irvine, and all subjects provided written informed consent for each study.

### 2.2. Saliva collection

All donors were asked to refrain from smoking, eating, drinking, or oral hygiene procedures for 30 min to 1 hour prior to samples collection. Saliva samples (n=93) were collected using the passive drool method according to previously established protocols. For the BEACoN cohort, saliva samples were collected between 7 am and 5 pm, and the exact time of sample collection was recorded. For the sleep study, saliva samples were collected within 1 hr of habitual wake time, which was between 6–11 am, and the exact time was recorded. Roughly two milliliters of unstimulated whole saliva was obtained. Samples were frozen at – 80C within 1 hour of collection. At the time of use, saliva samples were thawed and centrifuged (5000 g; 15 min; 4C) to remove mucins, insoluble material and cellular debris. Supernatants were collected and aliquoted for used in immunoassays.

### 2.3. Plasma collection

Blood was collected from sleep study participants within 1 hr of habitual wake time, which was between 6–11 am, with the exact time recorded. Blood was drawn via venipuncture from each participant into 7 mL lavender top EDTA tubes (BD 366450). Immediately after collection, each tube was gently mixed by inverting 8 to 10 times to ensure proper mixing of blood and anticoagulant, and then placed on wet ice. Blood samples were centrifuged in a swinging rotor bucket within 1 hour of collection at 2600 x RPM at 20°C for 10 minutes. The plasma samples were aliquoted by 0.750 mL increments into 2 mL polypropylene cryovials and transferred into a −80 °C freezer for storage until required for analysis.

### 2.4. PET

A*β* plaque burden was quantified using 18F-florbetapir (FBP) PET imaging on an ECAT High Resolution Research Tomograph (HRRT, CTI/Siemens, Knoxville, TN, USA) as described previously [20]. FBP PET data were available for n=86 individuals who also provided a saliva sample. Standardized uptake value ratios (SUVRs) were derived from data 50–70 minutes post-injection using a whole cerebellar reference region and 6 mm^3^ Gaussain smoothing. Native space regions of interest from FreeSurfer v.6.0.0 segmentations were used for regional FBP quantification. A cortical composite region described previously [21] as used as a measure of mean global FBP, using a threshold of >1.11 to determine A*β* positivity. In addition to mean global measures, mean SUVR values were determined for three composite regions representing early, intermediate, and late amyloid vulnerability, as identified in prior work [22] and described in a previous publication by our group [20]. For early A*β* accumulating regions, the composite region encompassed the precuneus, posterior cingulate, isthmus cingulate, insula, and both medial and lateral orbitofrontal cortices. The late-stage composite region was composed of the lingual, pericalcarine, paracentral, precentral, and postcentral cortices. Lastly, the intermediate stage region was defined as the remaining cortical areas [22].

### 2.5. Biomarker quantification

Levels of A*β*42, A*β*40 and A*β*38 were quantified in saliva and plasma samples participants using the V-PLEX A*β* Peptide Panel 1 (6E10) Kit 3-plex ECL immunoassay (Meso Scale Discovery (MSD), Gaithersburg, MD). Assays were run essentially according to MSD manufacturers protocol, with the exception of an extended incubation period and the inclusion of two additional low-end standards to try to better capture the low end of the standard curve. Saliva samples (25 ul) were diluted 1:2 in Diluent 35 (MSD), containing 1X Complete Protease Inhibitor (Sigma-Aldrich) and 1 mM EDTA. Whenever possible, samples were assayed after a single thaw to room temperature. On each platform, a single batch of reagents was used for all samples. Measurements were performed in duplicate, and sample measurements accepted if coefficients of variation (CV) across duplicates were less than 20 %. Concentrations (pg/ml) for each amyloid species were determined with MSD Discovery Workbench Software using curve fit models. Lower limits of detection (LLoD) were calculated as the concentration corresponding to the signal 2.5 times standard deviation above background and were 3.92, 0.63 and 0.12 pg/ml for A*β*38, A*β*40 and A*β*42, respectively. Intra-assay CVs were determined by taking the mean signal CV across each plate and were between 4.7–9.9 % for all markers. The inter-assay CV was determined using the mean concentration of three internal control plasma samples that were run on each plate and were 20.4 %, 5.88 % and 14.2 % for A*β*38, A*β*40 and A*β*42, respectively. Total protein levels in each saliva sample were determined using the BCA assays (Pierce^™^) according to previous studies [23]. In addition, blood contamination in saliva samples was assessed by measuring transferrin using an enzyme immunoassay kit (Salimetrics LLC).

### 2.6. APOE genotyping

DNA was isolated from saliva samples for genotyping assays. *APOE* genotypes were determined by a single nucleotide polymorphism (SNP) allelic discrimination assay using Taqman probes to the two *APOE*-defining SNPs, rs429358 (C_3084793_20) and rs7412 (C_904973_10) (ThermoFisher) and these were used to identify *APOE ε*2, *ε*3, and *ε*4 alleles. *APOE* genotypes were coded according to the presence (1) or absence (0) of any *ε*4 allele.

### 2.7. Cognitive assessments

Participants completed neuropsychological assessments including the Mini-Mental State Exam (MMSE), Montreal Cognitive Assessment (MoCA), the Rey Auditory-Verbal Learning Test (RAVLT) [24] and the Mnemonic Discrimination Tasks (MDT), with distinct assessments of object, spatial, and temporal mnemonic discrimination. For RAVLT, scores on the learning trials, immediate recall and delayed recall were assessed. For the object and spatial versions of the MDT, performance was measured using the lure discrimination index (LDI).

### 2.8. Statistical analyses

All statistical analyses were performed using RStudio R 4.3.1, IBM^®^ SPSS^®^ Statistics (version 25) or contchart.com. Raw data were first tested for normality using the Shapiro-Wilk normality test. Data were not normally distributed, hence associations to age and sex were carried out using Spearman correlation analysis and Mann-Whitney U tests, respectively. An outlier analysis was performed using Iglewicz and Hoaglin’s robust test for multiple outliers (two-sided test, modified Z score ≥ 3.5) using Ln-transformed data. One high outlier for A*β*38 and one high outlier for the A*β*42/40 ratio were detected and omitted for further analysis. No outliers were omitted when the A*β* species were normalized for total protein. Partial correlations relating A*β* biomarker data to global FBP SUVRs were carried out in SPSS using a non-parametric adjustment and were covaried for age, sex and time of saliva collection for saliva measures and age and sex for plasma measures. Salivary markers that were below the LLoD were not included in any analysis. A Bonferroni correction was applied to the results from our partial correlations analysis to adjust for multiple comparisons. The ability of salivary A*β* to identify A*β* PET status was determined using logistic regression models and receiver operating characteristic (ROC) curve analysis. Statistical models were adjusted for age, sex and time of sample collection according based on time since midnight.

## 3. Results

### 3.1. Participants and salivary Aβ measurements

This study involved cognitively unimpaired older adults with an average age of 70.1 yrs ± 6.6 yrs, a majority female (61.7 %) and predominantly white (88.4 %) (Table 1). Overall, 39.0 % of participants carried at least one *APOE4* allele and 35.3 % were considered to be A*β* PET-positive, determined using a 18F-florbetapir (FBP) global standardized uptake value ratio (SUVR) cut-off of 1.11 [21, 25] (Table 1).

Saliva levels of A*β*38, A*β*40 and A*β*42 were quantified in all subjects using the Mesoscale Discovery platform. The detection rates were less than 100 % for all A*β* peptides: 41.9 %, 78.4 % and 56.9 % for A*β*38, A*β*40 and A*β*42, respectively. The mean and median values for each are shown in Table 2. No A*β* peptide, nor the A*β*42/A*β*40 ratio showed a significant association with age nor sex (Table 2). Given that saliva samples were collected over the course the day in this study, we tested for associations to time of day of sample collection and found that A*β*42 did show a significant positive correlation with time of saliva sample collection (Table 2). Salivary A*β* species were not correlated with transferrin, a measure of blood leakage into the oral cavity, nor total protein levels, which can be used to assess sample concentration (Table 2). However, because some of the correlations to total protein were close to significance, we also normalized A*β* levels to the total protein in each sample in the analyses carried out below.

### 3.2. Associations between saliva and plasma Aβ and brain amyloid burden

**Fig. 1.**
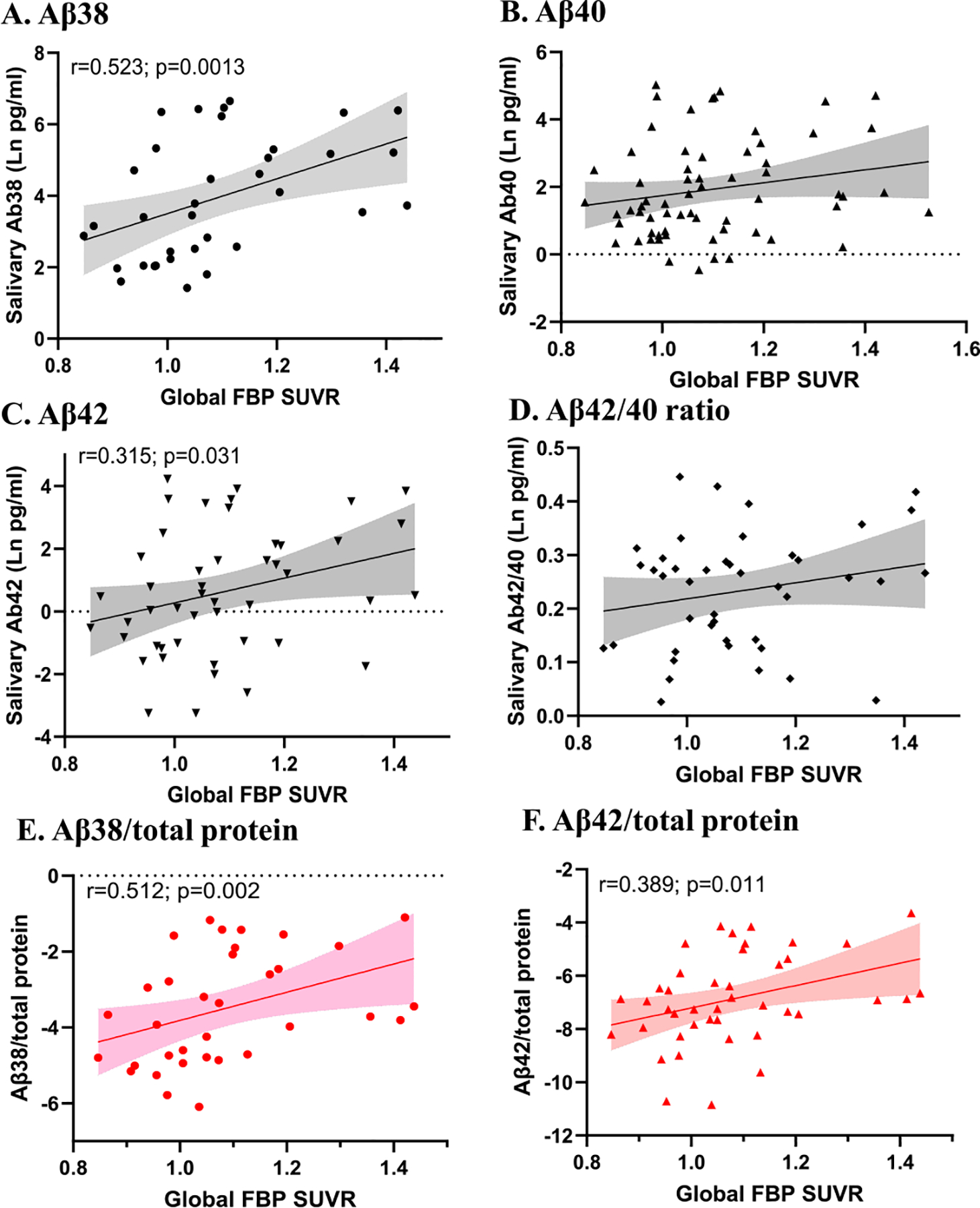
Correlations between salivary A*β* peptides and the mean18F-florbetapir (FBP) global standardized uptake value ratios (SUVR). Brain amyloid burden was determined by the use of the 18F-florbetapir (FBP) global standardized uptake value ratios (SUVR) considering the mean global SUVR for each participant. Spearman correlation analyses are shown with significant correlations as indicated. Panel A) shows A*β*38 (n=35), Panel B), A*β*40 (n=64), Panel C), A*β*42 (n=47 and Panel D), A*β*42/40 ratio (n=43). Panels E and F show A*β*38 and A*β*42 levels corrected by total protein.

To determine associations between salivary A*β* peptide levels and A*β* plaque burden, we compared levels of A*β*38, A*β*40, A*β*42 and the A*β*42/A*β*40 ratio with brain A*β*-PET data. Salivary levels of A*β*38 and A*β*42 were significantly positively correlated with global FBP SUVRs, before and after adjusting for age, sex and time of day of sample collection (Table 3; Fig. 1). The most robust correlation was observed for salivary A*β*38 (r=0.523; p=0.001, unadjusted and r=0.544; p=0.002, adjusted) (Table 3; Fig. 1). Significant correlations between salivary A*β*38 and A*β*42 and the mean global FBP SUVRs were also observed when saliva A*β* measures were corrected for the total protein in each sample (Fig. 1E and F). Focusing further on the time of day of sample collection, we assessed correlations between salivary A*β*38 and A*β*42 and the mean global FBP SUVRs only in subjects who had saliva collected during restricted hours in the morning (6 to 11 am, based on their habitual wake time (n=38)). Salivary levels of A*β*38 and A*β*42, but not A*β*40, were found to be significantly positively correlated with global FBP SUVRs (r=0.458; p=0.042; and r=0.421; p=0.057; for A*β*38 and A*β*42, respectively; Suppl Fig. 1).

Using plasma collected from the same BEACoN subjects, we previously found that plasma levels of A*β*38 and A*β*42 were negatively correlated with mean global FBP SUVR [17]. Herein, we validated this finding in participants from the sleep study, who provided blood samples time-matched with saliva samples. We observed that plasma levels of A*β*42, but not A*β*40, nor the A*β*42/40ratio, were negatively correlated with the mean global FBP SUVRs (Table 3). Plasma levels of A*β*38 were not measured in these participants, so this peptide could not be tested. From these time-matched blood and saliva samples, we were able to compare levels of A*β*40 and A*β*42 across the two fluids. We found that salivary and plasma levels of both A*β*40 and A*β*42 were significantly negatively correlated (r=–0.557; p=0.026; n=15 and r=–0.583; p=0.024; n=15, for A*β*40 and A*β*42, respectively; Suppl. Fig. 2). No significant correlations between plasma and saliva A*β* species were observed using samples collected across the day (i.e. from BEACoN participants), although the sample sizes for overlapping plasma and saliva samples were also low n=16, n=41 and n=25 for A*β*38, A*β*40 and A*β*42, respectively (Suppl Table 1).

To further characterize the extent of the associations between salivary A*β* peptide levels and A*β* plaque burden, we investigated associations between salivary A*β* and A*β* plaque burden in different regions of the brain according to timing of A*β* accumulation. Although there is not a complete consensus on which areas are affected earliest by A*β* deposition, we used composite regions which represent cortical regions vulnerable to A*β* accumulation in early, intermediate, and late stages of AD according to Mattsson and colleagues [22]. Mean FBP SUVRs were obtained for early, intermediate, and late-accumulating regions per previous studies from our group [20]. We found that A*β*38 showed the most robust correlation with FBP SUVRs from early-accumulating brain regions, although significant correlations were observed for A*β*38 and FBP SUVRs from all regional composites (Table 4). A*β*42 showed similar correlations as A*β*38, but with less statistical significance, and A*β*40 levels and the A*β*42/A*β*40 ratio showed significant correlations only with FBP SUVRs from late-accumulating brain regions (Table 4).

### 3.3. Salivary Aβ38 levels can predict Aβ-PET positivity

**Fig. 2.**
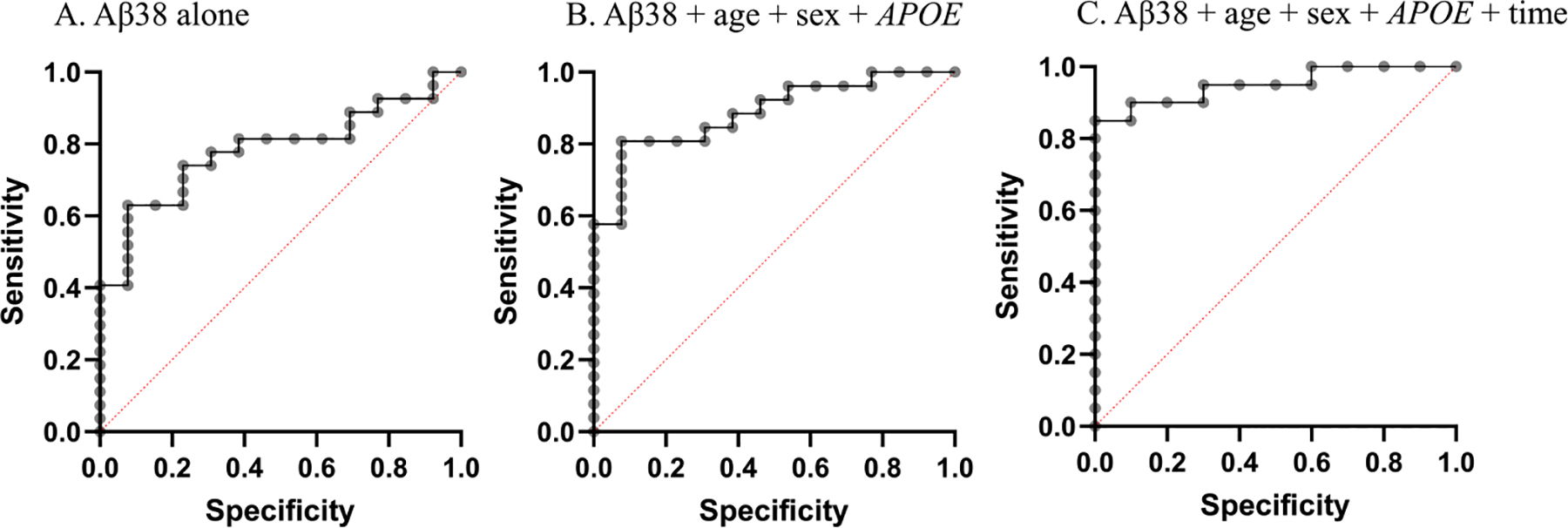
ROC curves for A*β*38 to predict brain amyloid PET in cognitively normal participants. Brain amyloid positivity was determined mean18F-florbetapir (FBP) global standardized uptake value ratios (SUVR) using a cut-off of 1.11. The curves reflect the data shown in Table 5.

We next examined how well salivary A*β*38 levels could predict A*β*-PET positivity based on a mean global FBP SUVR using a threshold of > 1.11 SUVR [21, 25]. Using receiver operating characteristic (ROC) curves to evaluate the performance of a logistic regression model, we found that the area under the curve (AUC) for A*β*38 alone to predict amyloid positivity was 0.784 (95 % CI, 0.642 to 0.925, p=0.0041), while including age, sex and *APOE* genotype in the model improved the AUC to 0.887 (95 % CI, 0.785 to 0.989, p<0.0001) (Table 5, Fig. 2). Interestingly, including time of sample collection strengthened the model to an AUC of 0.950 (95 % CI, 0.876 to 1.00, p<0.0001) (Table 5). A*β*42 alone or in combination with age, sex and *APOE* genotype was less sensitive in predicting A*β* positivity (Table 5).

### 3.4. Salivary Aβ levels are not associated with performance on sensitive cognitive tasks

Given the association between salivary A*β*38 and A*β*42 and FBP SUVR in brain regions known to accumulate A*β* early, we tested whether A*β* levels were associated with early memory measures, including the RAVLT and MDT in cognitively unimpaired older adults. No significant associations were observed between salivary levels of any A*β* petide and RAVLT learning, immediate or delayed recall, nor the combined LDI from the spatial and object MDT tasks (data not shown). Similarly, no significant associations were detected between the mean global FBP SUVRs and any RAVLT or MDT LDI measures (data not shown).

## 4. Discussion

In this study, we demonstrated that salivary levels of A*β*38 and A*β*42 are significantly, positively correlated with brain A*β* burden and that salivary A*β*38, in particular, can predict A*β* positivity in cognitively normal older adults. Detecting A*β* pathology early in the disease process has been an important goal in the diagnosis and management of AD, as well as for selection of patient for clinical trials with amyloid-lowering compounds. Given the need for more widely-accessible, non-invasive biomarkers in this context, our studies suggest that salivary A*β*38 and/or A*β*42 could potentially meet this objective.

Our current findings add to a growing body of literature demonstrating quantification of A*β*42 and A*β*40 in saliva samples, and in particular, studies reporting significant associations to AD. Past studies using ELISA have shown that levels of salivary A*β*42 were higher in AD patients compared to non-demented controls and those with non-AD dementias [12,15]. Other studies showed increased levels of A*β*42 in patients with MCI 13,26, including results showing that the salivary levels of A*β*42 increased according to disease severity [14]. One study even reported diagnostic accuracy for salivary A*β*42 to discriminate AD patients from controls (area under the curve=0.81) [13]. In two of the studies showing increases in salivary A*β*42 in AD, levels of A*β*40 were also measured in saliva, but did not show significant differences in AD patients compared to controls [10,11].

In addition to showing correlations between A*β* peptides and A*β*-PET measures, our findings are novel in that they are the first to report salivary levels of the A*β*38 peptide, which we show is more strongly correlated with A*β*-PET than A*β*42. Correlations for A*β*38 and A*β*42 were also significant when analyte levels were corrected for total protein, which we do not believe is necessary for salivary biomarker clinical utility, but is one approach that has been taken in past studies [27]. Total protein can be useful to assess the concentration of the sample, which can occur with reduced saliva flow. For example, some medications can reduce the output of saliva (i.e. reduced flow-rate), which might result in a more concentrated sample. Also, it has been shown that unstimulated salivary secretion is impaired in AD patients [28,29], hence once possibility from previous studies was that A*β* levels were higher due to decreased saliva production resulting in more concentrated saliva. However, we did not detect significant correlations between saliva A*β* species and total protein measurements and given that the A*β* correlations to FBP SUVR remained highly significant when value were corrected for total protein, argues against a reduced flow-rate contributing to the observed effect.

Many salivary analytes are known to show diurnal variations, for example, salivary cortisol, which exhibits peak levels observed upon wakening [30]. Accordingly, we observed that A*β*42 were significantly associated with time of day of the sample collection, with levels increasing throughout the day. These findings are consistent with other reports providing evidence of diurnal fluctuations in A*β*40 and A*β*42 levels in both plasma and CSF samples, with levels increasing in the evening compared to morning [31–33]. Surprisingly, A*β*38 and A*β*40 did not show similar relationships. Currently, the factors driving this variation across the day are unknown, but could be related to sleep quality, circadian factors, exercise, or even food intake [33,34]. In the BEACoN study, the timing of sample collection was not restricted, hence we included the time of sampling in our analyses as a covariable. However, we were able to validate our findings in a second cohort of subjects, where morning collection times were restricted to 6–10 am. Overall, this work implies that the time of day of sample collection is important in the interpretation and implementation of fluid biomarkers in AD research and care; therefore, it is highly recommended to collect saliva samples within a restricted time frame to avoid potential diurnal variations in biomarker levels.

The findings that salivary A*β* levels are positively correlated with brain A*β* burden is opposite to what we and others have previously observed with plasma A*β* levels, whereby plasma A*β*40 and A*β*42 have been shown to be negatively associated with brain A*β* burden 18,35,36. Similar negative associations have been shown for CSFA*β*42 levels and the A*β*42/40 ratio [37,38], and given the known correlation between A*β*42 levels in CSF compared to blood 18,35, this is not surprising. In this study, we found that salivary and plasma levels of A*β*42 were significantly negatively correlated, however, one limitation of this finding is the small sample size (n=15), due to the low detection rates for salivary A*β*42 and the limited number of participants from whom blood was collected. Interestingly, Boschi and colleagues demonstrated a significant negative correlation between salivary and CSF A*β*42 concentrations (r = −0.562, p < 0.001) [9]; however, it should be noted that another study did not report any correlations between saliva and CSF A*β*42 [39]. Combined, these findings are consistent with the directions of the correlations we observed in this study.

A major question exists regarding why levels of A*β* in saliva track in the same direction as A*β* plaque formation in the brain, when blood and CSF levels do not. The mechanism by which this occurs is not certain clear. One consideration is that A*β*38 plays a different role in AD compared to A*β*42 or other A*β* species. Previous studies have shown that the A*β*38 peptide does not exhibit toxicity *in vivo*, nor does it accumulate into plaques and research suggests that it can even inhibit A*β*42-associated aggregation [40,41]. In our study, the correlations to FBP SUVRs were the strongest for A*β*38, suggesting that this peptide may reflect a different mechanism than A*β*42. In this case, salivary A*β*38 levels may originate from the cranial nerves innervating salivary glands or CSF leakage via exiting nerves. Specifically regarding A*β*42, one possibility is that the saliva effect is a peripheral phenomenon, reflecting the notion that AD is a whole body disease. It is known that accumulation of A*β* occur in other tissues besides in the brain, including the nasal mucosa [42], and the lacrimal [43] and salivary glands [44] and in salivary epithelial cells [45]; hence, A*β* peptides could be released, directly or indirectly into saliva from these sources in a manner that tracks with pathology in the brain.

Over the past decade, saliva has been growing in interest as a biofluid with meaningful diagnostic potential. However, a main drawback to the use of saliva for biomarker studies is the lack of standardization of saliva collection, processing and lack of consideration of pre-analytical and post-analytical parameters, which can hinder comparisons across laboratories and studies. For example, assessing saliva sample quality is important, especially with regards to blood contamination, which can come from gingivitis, or open sores in the mouth, and can be important when assessing biomarkers whose concentrations are higher in blood compared to saliva. In our study, transferrin was used to assess samples for blood contamination, whereby it has been recommended that samples over 0.5 mg/dL are omitted for analysis. Only one of our saliva samples had a value of 0.5 mg/dL (i.e. 9.03 mg/dL), but this sample did not give detectable values for A*β*38 or A*β*42 so was not included in any analyses.

Our study is not without limitations. A main drawback is the small number of biomarker measurements that were available for analysis. Despite the cohort size of n=86 participants, detection rates for the A*β* species were low, especially with regards to A*β*38, which showed the lowest detection rate (41.9 %). While detection rates for A*β*40 and A*β*42, were slightly better (78.4 % and 56.9 % for A*β*40 and A*β*42, respectively), all A*β* species were well under 100 % detection. While part of the low detection rates could be due to low abundance of these biomarkers in saliva samples, it is also possible that secondary structure, or other oligomeric forms of A*β* peptides, in the saliva matrix can result in the assay antibodies not fully recognizing all A*β* protein. An argument against the undetected samples reflecting levels too low to be quantified is that there was no relationship between the numbers of undetected samples from individuals who were A*β*-PET (+) compared to those who were A*β*-PET(−) (Fisher’s Exact test, p=0.65–1.0; Suppl. Table 2). Clearly, improved assays to quantify A*β* species in saliva are needed in order to overcome this drawback in future studies. Further, given the small sample size of this study, selection bias is possible, whereby our findings may not be applicable to other populations. Additional studies will be needed to address this issue.

It is worth mentioning that other AD biomarkers, such as YKL–40, neurofilament light (NfL), glial fibrillary protein (GFAP) and total tau protein have been quantified in saliva samples and have shown to be readily detected [13,26,46]. In particular, our previous studies assessing these biomarkers in saliva samples from Huntington’s disease patients observed detection rates of 100 %, 88.4 %, 81.1 % and 98.9 %, for YKL40, NfL, GFAP and total tau, respectively [47].

Also in this study, we have carefully considered the timing of sample collection, concentration of the saliva sample and stabilization of protein by the addition of protease inhibitors to the samples, all of which can help to standardize saliva methodology. However, the fact that multiple studies, using different approaches and methods have reported increases in A*β*42 in AD, demonstrates the validity of salivary A*β* as a potential non-invasive biomarker for AD.

There is a pressing need for accessible and inexpensive biomarkers for AD diagnosis and to facilitate widespread screening, particularly in underserved groups. Our current studies suggest that salivary A*β*38 and/or A*β*42 could represent a non-invasive, and more widely accessible biomarker, for clinical utility in AD. Nonetheless, standardized saliva protocols must be established before these biomarkers can be routinely used for AD diagnosis and monitoring. Additionally, longitudinal studies are necessary to establish the reliability and predictive value of saliva-based biomarkers in different stages of AD.

## Supplementary Material

Supplement

Supplementary material associated with this article can be found, in the online version, at doi:10.1016/j.tjpad.2025.100216.

## Figures and Tables

**Table 1:** Summary of participants used in this study.

	Males:	Females:	Total:
Number	41	57	93
Mean age in years (S.D.)	70.1 (6.49)	70.0 (6.65)	70.0 (6.58)
Mean Edu (S.D.)	17.2 (1.91)	16.1 (2.23)	16.5 (2.35)
Mean MMSE (S.D.)	28.6 (1.27)	28.5 (1.40)	28.6 (1.34)
Race (White)	90.0 %	87.2 %	88.40 %
ApoE4(+)	48.7 % (+)	32.7 % (+)	39.0 %(+)
Amyloid PET (+)	28.2 % (+)	40.0 % (+)	35.3 %(+)

The number of subjects include those with available PET imaging data (n=86) plus n=7 subjects who provided a saliva sample, but did not undergo PET imaging. Edu, Education; MMSE, Mini-mental state examination; ApoE, apolipoprotein E; 18F-florbetapir positron emission tomography (PET) was used to determine amyloid positivity with an 18F-florbetapir (FBP) global standardized uptake value ratios (SUVR) cut-off of 1.11.

**Table 2:** Summary of amyloid beta (A*β*) 38, 40, 42, and 42/40 ratio levels in saliva samples from cognitively unimpaired individuals.

	A*β*38:	A*β*40:	A*β*42:	A*β*42/40:
**N:**	39	73	53	50
**% Detection:**	41.9	78.4	56.9	53.7
**Mean ± S.D.:**	159.5 ± 226.5	19.32 ± 34.18	8.42 ± 15.12	0.283 ± 0.312
**Median (range):**	42.70 (4.13–772.3)	4.49 (0.630–153.1)	1.41 (0.039–68.24)	0.264 (0.026–2.30)
**Age (r; p-value):**	−0.192; 0.241	0.047; 0.692	−0.137; 0.328	−0.128; 0.375
**Sex (p-value):**	0.724	0.693	0.553	0.96
**Collection time**^1^ **(r; p-value):**	0.172; 0.348	0.082; 0.534	**0.323; 0.034**	0.255; 0.108
**Total protein (r; p-value):**	0.282; 0.086	0.201; 0.111	0.271; 0.059	0.022; 0.884
**Transferrin**^2^ **(r; p-value):**	0.415; 0.077	0.016; 0.917	0.315; 0.096	0.304; 0.123

N=number of samples measured within the detection limit from a total number of n=93 subjects who provided a saliva sample. For the A*β*42/40 ratio, there were three samples that gave a detectable A*β*42 value, but not for A*β*40. S.D., standard deviation. Age, collection time, total protein and transferrin associations were determined using Spearman correlation analysis. Sex associations were determined by Mann-Whitney U test.

1 Sample collection times were available for n=32, n=60 and n=43, for A*β*38, A*β*40 and A*β*42, respectively.

2 Transferrin was only measured in BEACoN samples, hence, the overlap of samples with both transferrin and detectable A*β* was n=19, n=44 and n=29, for A*β*38, A*β*40 and A*β*42, respectively.

**Table 3:** Unadjusted and adjusted correlations for the association between saliva and plasma amyloid beta peptides and mean global brain amyloid burden.

Number:	Saliva				Plasma		
	A*β*38: 35	A*β*40: 64	A*β*42: 47	A*β*42/40: 43	A*β*40: 40	A*β*42: 40	A*β*42/40: 40
Unadjusted Rho:	**0.523**	0.213	**0.315**	0.186	−0.341	**−0.516**	−0.265
Unadjusted p-value:	0.001*	0.092	**0.031**	0.228	0.103	0.01*	0.211
Adjusted Rho:	**0.544**	0.2	**0.32**	0.177	−0.321	**−0.504**	−0.25
Adjusted p-value:	0.002*	0.135	**0.044**	0.288	0.145	**0.017**	0.262

Brain amyloid burden was determined by the use of the 18F-florbetapir (FBP) global standardized uptake value ratios (SUVR) considering the mean global SUVR for each participant. Amyloid beta (A*β*). Correlations were adjusted for age, sex and saliva collection time for saliva and for age and sex for plasma values (n=40). Significant correlations are shown in bold. Asterisk denotes significant finding after Bonferroni correction.

**Table 4:** Partial correlations for the association between amyloid beta peptides and brain amyloid from brain regions associated with different stages of disease.

Number		A*β*38: 35	A*β*40: 64	A*β*42: 47	A*β*42/40: 43
Early Stage	Rho	**0.510**	0.231	**0.385**	0.249
	p-value	<0.001*	0.056	**0.009**	0.112
Intermediate Stage	Rho	**0.467**	0.242	**0.316**	0.161
	p-value	0.002*	0.045	**0.034**	0.309
Late Stage	Rho	**0.419**	**0.257**	**0.38**	**0.323**
	p-value	0.006*	**0.033**	**0.01**	**0.037**

Partial correlations were adjusted for age and sex, and were run with a non-parametric adjustment. Bold font denotes statistically significant correlation. Asterisk denotes significant finding after Bonferroni correction. For early-stage A*β* accumulation, the composite region encompassed the precuneus, posterior cingulate, isthmus cingulate, insula, and both medial and lateral orbitofrontal cortices. The late-stage region was composed of the lingual, pericalcarine, paracentral, precentral, and postcentral cortices. Lastly, the intermediate stage region was defined as the remaining cortical areas.

**Table 5:** Receiver operating characteristic (ROC) models predicting brain amyloid positivity in cognitively normal participants.

Model:	AUC:	SE:	95 % CI:	P-value:
1. Age + Sex	0.623	0.061	0.502 to 0.743	0.042
2. Age + Sex + APOE	0.732	0.052	0.630 to 0.834	**0.0002**
3. A*β*38 alone	0.784	0.072	0.642 to 0.925	**0.0041**
4. A*β*42 alone	0.649	0.083	0.487 to 0.812	0.078
5. Age + Sex + APOE + A*β*38	0.887	0.052	0.785 to 0.989	**< 0.0001**
6. Age + Sex + APOE + A*β*42	0.794	0.063	0.669 to 0.919	**0.0024**
5. Age + Sex + APOE + A*β*38 + time of collection	0.95	0.037	0.876 to 1.00	**< 0.0001**

Brain amyloid positivity was determined mean18F-florbetapir (FBP) global standardized uptake value ratios (SUVR) using a cut-off of 1.11.
