# Supplementary material for: Salivary levels of amyloid beta reflect brain amyloid beta burden in cognitively-normal older adults
Source: J Prev Alzheimers Dis. 2025 Jun 9;12(7):100216. doi: 10.1016/j.tjpad.2025.100216 (PMC12252581; doi:10.1016/j.tjpad.2025.100216)
Supplement: Supplementary file 1 [file mmc1.docx]

**Suppl. Figure 1. Correlations between salivary Aβ38 and Aβ42 and the mean18F-florbetapir (FBP) global standardized uptake value ratios (SUVR) in a subset of individuals with restricted saliva collection times.**

Saliva samples were collected between the hours of 6-11 am. Pearson correlation analysis shows a significant correlation between mean FBP SUVR and Aβ38 (n=20 subjects) and a close to significant correlation for Aβ42 (n=21 subjects). The numbers of data points reflect the number of detected measurements out of a total of n=38 subjects.

**Supplementary Figure 2. Correlations between salivary and plasma Aβ40 (A) and Aβ42 (B) in a subset of the sleep study participants whose saliva and plasma was collected between the hours of 6-10 am.**


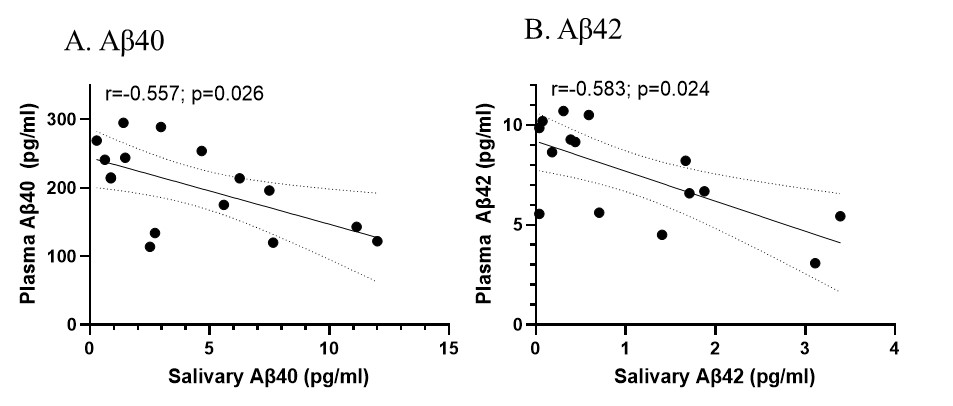


Saliva samples were collected between the hours of 6-11 am. Pearson correlation analysis shows a significant correlation between plasma and saliva Aβ40 and Aβ42, as shown. Data points reflect only those levels above the detection limit, n=15 for both Aβ40 and Aβ42 for this subset.

**Supplementary Table 1. Correlation matrix showing relationships between Aβ peptides in blood samples (“B”) versus saliva (“S”).**

|  | **B_Aβ38** | **B_Aβ40** | **B_Aβ42** | **S_Aβ38** | **S_Aβ40** | **S_Aβ42** |
| --- | --- | --- | --- | --- | --- | --- |
| **B_Aβ38** |  | 7.74E-06 | 3.36E-14 | 0.131 | 0.473 | 0.141 |
| **B_Aβ40** | 7.74E-06 |  | 1.33E-09 | 0.168 | 0.06 | 0.122 |
| **B_Aβ42** | 3.36E-14 | 1.33E-09 |  | 0.144 | 0.286 | 0.058 |
| **S_Aβ38** | 0.131 | 0.168 | 0.144 |  | 6.40E-15 | 5.02E-18 |
| **S_Aβ40** | 0.473 | 0.0602 | 0.286 | 6.40E-15 |  | 6.26E-13 |
| **S_Aβ42** | 0.141 | 0.122 | 0.058 | 5.02E-18 | 6.26E-13 |  |

Numbers reflect the p-values from a Spearman correlation analysis. Numbers of overlapping samples for each Aβ compared to itself between the two biological fluids was n=16, n=41 and n=25 for Aβ38, Aβ40 and Aβ42, respectively.

**Suppl. Table 2. Comparison of the detection rates for each Aβ species and Aβ-PET positivity.**

“0” reflects Aβ-PET (-) individuals and “1” denotes Aβ-PET (+) individuals. The p-values reflect the results of Fisher’s Exact test for each peptide. Note, the total numbers of samples below is different from Table 2, given that there were n=7 additional saliva samples from individuals who did not undergo PET imaging
